# THE SOCIAL DETERMINANTS OF BRAIN HEALTH: FROM THE SEARCH FOR MECHANISMS TO RECOMMENDATIONS TO INCREASE EQUITY

**DOI:** 10.1093/geroni/igad104.0226

**Published:** 2023-12-21

**Authors:** Anja Leist, Graciela Muniz-Terrera

## Abstract

Dementia risk has been established to be partly attributable to modifiable social and behavioral risk factors. However, the biological pathways behind these associations and the effects of intervening on present risk factors to reduce dementia risk have not been fully elucidated. The symposium will present latest research on the social determinants of brain health and dementia. We will explore proposed mechanisms between modifiable risk factors and risk of dementia, and present findings on the modifiability of dementia risk through addressing risk factors. Specifically, the first presentation reports analyses testing possible pathways from socioeconomic status, notably childhood and adulthood indicators such as (parental) education and occupation, to cognitive functioning in later mid-adulthood via indicators of brain damage and white matter connectivity with the Maastricht Study. The second presentation reports empirical analyses testing the possible mediating role of the gut microbiome in the relationships between social and modifiable risk factors and Mild Cognitive Impairment with data of the controls of the Luxembourg Parkinson’s Study. The third presentation reports on the associations between hearing loss as a modifiable risk factor and incident dementia in the UK Biobank and investigates the potential to modify hearing loss-associated dementia risk through the use of hearing aids. Lastly, the fourth presentation moves to recommendations for policy and government action. It gives an overview of various fields in which policy and legislation could implement cognitive impact assessments to protect individuals’ cognition, with the aim of improving population brain health and health equity. This is a Brain Interest Group Sponsored Symposium.

